# Men’s Migration, Women’s Personal Networks, and Responses to HIV/AIDS in Mozambique

**DOI:** 10.3390/ijerph10030892

**Published:** 2013-03-06

**Authors:** Winfred Avogo, Victor Agadjanian

**Affiliations:** 1 Department of Sociology and Anthropology, Illinois State University, Campus Box 4660, Normal Illinois, IL 61790, USA; 2 T. Denny Sanford School of Social and Family Dynamics, Arizona State University, Tempe, AZ 85287, USA; E-Mail: victor.agadjanian@asu.edu

**Keywords:** labor migration, personal networks, HIV/AIDS, social capital, homophily, selection, social influence, health, sub-Saharan Africa, Mozambique

## Abstract

This study brings together the literature on social network approaches to social capital and health and on migration and HIV risks to examine how non-migrating wives of labor migrants use their personal networks to cope with perceived risks of HIV infection in rural southern Mozambique. Using data from a 2006 survey of 1,680 women and their dyadic interactions, we compare the composition of personal networks, HIV/AIDS communication, and preventive behavior of women married to migrants and those married to non-migrants. Results show that migrants’ wives were more likely than non-migrants’ wives to have other migrants’ wives as personal network members, to engage in HIV/AIDS communication, and to discuss HIV prevention. However, they were no more likely to talk about HIV/AIDS with migrants’ wives than with non-migrants’ wives. They were also no more likely to talk about AIDS and its prevention than non-migrants’ wives who express worry about HIV infection from their spouses. Finally, we detect that network members’ prevention behavior was similar to respondents’, although this did not depend on migration. We contextualize these findings within the literature and discuss their policy implications.

## 1. Introduction

In the last two decades, the public health literature has devoted considerable attention to the impact of social capital on health behavior and outcomes. Although social capital has been conceptualized, measured and applied differently, the sociological literature tends to see social capital primarily as valued resources (such as social support, information, *etc.*) embedded within and gained through membership in extra familial personal networks (network approach) [1,2,3,4]. In this study, we apply the network approach to social capital to examine how resources embedded within egocentered personal networks may facilitate communication about HIV/AIDS and prevention behavior.

The second body of knowledge that our study engages comes from the epidemiological and sociological literature that has identified migration as an important factor fueling the HIV/AIDS epidemic, especially in sub-Saharan Africa. Although the specific pathways through which migration impacts HIV/AIDS remain disputed, studies typically suggest that people who are more mobile or frequently change residence and their partners are at a higher risk of HIV infection and other sexually transmitted diseases (STDs) than people in more stable living conditions [5,6]. 

However, these two bodies of literature have remained largely separate. The literature on migration and HIV risks, for example, has paid relatively little heed to research that has documented how interactions within personal networks may offer guidance and support for individual assessment of risks and exposure to HIV infection [7,8]. Similarly, although the literature on social capital and health has emphasized the role of social support networks as a health advantage of migrants in places of migration destination [9], not much research exists on the role of migration related personal networks in HIV/AIDS communication and prevention. Furthermore, work on migration and HIV risks has focused primarily on migrants’ vulnerability in places of migration destination and only a few studies have examined the vulnerability of migrants’ partners and the role of their personal networks in reducing the risks of infection in areas of migration origin [10]. 

Our study aims to bridge these two bodies of literature by using the network approach to social capital to investigate how left-behind women in rural Mozambique engage their personal networks to cope with risks of HIV infection. Specifically, we focus on the content of communication on HIV/AIDS and prevention behavior and the role of homophily, selection and social influence in the personal networks of wives of migrants and wives of non-migrants. 

## 2. Background

Less public health research has integrated a network approach to social capital and health. Much of the work on social capital and health has been focused on social cohesion and influence, trust within a community, norms of reciprocity and social support [11]. For example, one of the earliest applications of social capital to health found evidence that income inequality within a community was related to reduction in group membership and social trust which in turn was associated with increased rates of mortality [12]. Another study emphasized contextual influences of the collective exerted on individuals that affect their health [13]. Other research drawing on political scientist Robert Putnam’s [14,15,16] community-level conceptualization of social capital has found that stronger social relationships and social support within a community are associated with better health outcomes either directly or indirectly through stress reduction behavior [13,17] and that perceived adequacy of support is more important than received support [18,19].

Although research on social capital and health in Sub-Saharan Africa is scarce, the few studies that have applied social capital to health outcomes such as HIV risks have done so at the community level and not as resources embedded within personal networks as originally proposed by Pierre Bourdieu [1] and built upon by Lin [3]. For example, a cross-sectional study in Zimbabwe found that membership and participation in local community groups is associated with risk-avoiding behavior [20]. Another study using a similar conceptualization of social capital suggests that different types of social capital (structural and cognitive) have potential benefits for HIV prevention through participation in formal social institutions [21]. A more recent study examined how migration acts as a conduit for HIV transmission in South Africa and proposed using social capital theory, a prevention model that mobilizes community leaders, institutions and stakeholders to combat AIDS [22]. 

Consistent with Bourdieu’s definition of social capital (see [1] pp. 248–249), network-based approaches to social capital and health consider social capital as network-mediated benefits that can be drawn on by individual group members for health promotion and maintenance. This conceptualization of social capital is important because it highlights unequal access to network-based resources as network members may differ in terms of the composition of their social networks, the different social positions occupied by network members and by the exclusion of outsiders [4]. It also goes beyond emphasis on the beneficial aspects of social capital, as portrayed in Putnam’s conceptualization of social capital to include its negative and contradictory effects. Using Bourdieu’s conceptualization for example, Rankin [23] highlights how the practice of gifting and reciprocity among unequals in Nepal creates affective bonds that conceal the hierarchical nature of social relationships. Specifically, gifts (not matched by counter gifts) to low-caste inferiors by their high-caste superiors conceal abuses inherent in the caste system by creating lasting bonds of trust and obligation that restrict the freedoms of lower-caste. Thus Bourdieu’s social capital theory warns of negative consequences of social capital in what he refers to as “symbolic violence” that binds the oppressed to their oppressors through norms of reciprocity [23,24]. In other research on the negative aspects of social capital generated within social networks, a study on teenage pregnancy in a Baltimore ghetto found that dense but truncated kinship networks in ghettos simultaneously deprived members access to information and opportunities outside the network while promoting cultural lifestyles that condoned teenage pregnancy as a means to gain adult status and independence [25]. 

Overall, when network-based approaches are applied to health outcomes, they often measure social capital by using two main techniques: (a) egocentric networks obtained through a name generator technique which uses survey questions to ask respondents (egos) to nominate others in their personal network (alters) who are important to them and who provide access to resources such as advice, prestige, social status and social and political connections; and (b) Whole networks obtained through saturation survey techniques which maps everyone within a defined social structure or social network [2,3]. Both techniques are important to fully understand the impact of social capital on health. However, egocentered networks are more common in the literature due to relatively less stringent data demands [26]. 

Although men’s labor migration has been associated with risks of HIV infection for non-migrating rural partners, research on HIV serodiscordance among couples also point to migrant men who return to partners already infected [27]. The dynamics of labor migration in Southern Africa thus offers a unique avenue to contribute to the literature on social capital and health by examining the role of spousal migration in the composition of egocentered networks (hereafter also personal networks) and the use of network-based resources in communicating and countering the threat of HIV/AIDS both within and outside the marriage. In this study, we draw on the association of migration with risks of HIV infection to examine the social support systems women develop through their personal networks to respond to such risks by linking network concepts of selection, homophily, and social influence to the literature on social capital and health. 

## 3. Conceptual Framework

To conceptualize the association between men’s labor migration and migrants’ wives use of resources embedded within personal networks to cope with the risk of HIV infection; we draw on the embedded resources conceptualization of social capital proposed by [3]. 

We specify how labor migration of men affects their wives’ opportunities to construct and mobilize network-based resources and how the composition of their personal network may facilitate or constrain access to resources to reduce the risk of HIV infection. For example, previous literature on network theory has shown that personal network members are not chosen at random but systematically through homophily, *i.e.*, the tendency for individuals to form ties with those who are similar to them [28,29]. Thus homophilous personal networks facilitate the formation of expressive ties based on interpersonal attraction and these ties can be mobilized and shared with network members to preserve physical health [3]. Network approaches to health are especially important for research and a comprehensive strategy for responding to the AIDS crisis in Africa. Given the limitations of traditional health education approaches which are based mainly on conscious rational choice by individuals, highlighting how on-going face-to-face social interaction occurs among migrants’ wives may help to better understand how HIV risks and worry of infection is interpreted and how prevention programs could be disseminated to target specific groups and the general population as part of a comprehensive strategy to combat AIDS. 

Applying insights from network approaches on social capital to migration and health, we argue that network characteristics and resources will not only enable migrants’ wives to express worries and cope with heightened fears of HIV infection associated with labor migration but the structure of personal networks particularly homophily, selectivity and social influence that may be generated within these networks will be important for HIV/AIDS-related communication and for HIV testing and prevention. Based on this broad prediction, we conceptualize and test specific hypotheses on how migrants’ wives engage network resources as a way of coping with worries associated with HIV infection. 

First, while men’s labor migration entails benefits for left-behind women’s socio-economic status and autonomy [30], it is also said to generate psychological strain among women [31,32]. In settings such as rural southern Mozambique, where labor migration is common and is usually accompanied by notions of increased risk of HIV infection, migrants’ wives may tend to interact with women in similar circumstances and with similar characteristics either by choice or by virtue of ties between their respective migrant partners. Thus differences in the composition of network members of migrants and non-migrants may affect access to resources that can be mobilized to preserve physical health.

Second, due to perceived vulnerability of migrants’ wives to HIV infection, and general stigmatization of women as vectors of the disease [33], membership in personal networks and access to social resources embedded within personal networks (such as the willingness of network members to loan money to each other and other attributes such as being of same religious denomination, age and kin ties) may help build trust and facilitate communication about HIV/AIDS. Conversations on AIDS will provide migrants’ wives an avenue to share opinions about the disease, assess risks of HIV infection, overcome stigma that is still associated with the disease and explore options for prevention. Conversely, we argue that non-migrants’ wives may not feel as vulnerable to HIV infection; hence network resources may not be mobilized to engage in as much communication about HIV/AIDS as in the case of migrants’ wives.

Third, because close kin and friends often form confidant networks which may be supportive and encouraging of health related behaviors, conversations on HIV/AIDS may be selective on spouse’s migration status. Lastly, given perceptions of an increased risk of HIV infection associated with labor migration, we expect the content of migrants’ wives HIV/AIDS-related conversations to emphasize prevention of HIV infection rather than other HIV/AIDS-related topics.

In the second part of our conceptual framework, we focus on HIV prevention and testing behavior among migrants’ and non-migrants’ wives. Broadly, we draw from the social interaction and family planning and HIV communication literature which, helps explain how individual risk behavior is influenced by social norms and prevention beliefs shared among network members [34]. Thus personal network studies that focus on sexual and reproductive behavior have found similarities between the behavior and characteristics of network members and those of egos. For example, a study found an association between the specific methods of contraception used by egos and those used by their personal network members [35]. Similarly, another study concluded that men’s extra-marital sexual behavior was associated with that of their best friends and friends with whom they talk about AIDS [36]. Thus broadly speaking, we expect network member’s reported HIV prevention and testing behavior to be associated with wives’ behavior. Applying this conceptualization to migration status, we expect prevention and testing behavior of migrants’ wives to be selective on both the migration status of their spouses and on the migration status of the partners of their network members as well as on their network members’ prevention and testing behavior. 

Overall, our conceptual framework yields the following specific hypotheses:

- **H1**: Migrants’ wives are more likely to have fellow migrants’ wives as personal network members than are non-migrants’ wives, net of other characteristics.
- **H2**: Migrants’ wives are more likely to use embedded resources within their networks to engage in communication about HIV/AIDS with members of their personal network than are non-migrants’ wives, net of other characteristics.
- **H3**: Migrants’ wives are more likely to use embedded resources within their networks to converse about HIV/AIDS with network members who are also migrants’ wives than with network members who are not migrants’ wives, net of other characteristics.
- **H4**: Migrants’ wives are more likely to use embedded resources within their networks to discuss HIV prevention in their conversations with network members compared to non-migrants’ wives, net of other characteristics.
- **H5**: Migrants’ wives are more likely to have been tested for HIV and to use HIV prevention if their network members are also migrants’ wives and have tested for HIV and used prevention, net of other characteristics.

## 4. Study Setting

Data for this study come from a survey conducted in Mozambique, a country in southeast Africa with a population of some 23.7 million [37]. Like its southern African neighbors, Mozambique is located in the continent’s “AIDS belt” which is mostly concentrated in 16 contiguous countries in eastern and southern Africa stretching from Djibouti and Ethiopia through the east side of the continent to South Africa. Together these countries account for more than 50 percent of worldwide HIV infections [38]. 

In Mozambique, the national prevalence rate among adults aged 15–49 increased from 8.2 per cent in 1998 to 16.2 per cent in 2004 [39], putting that country at the 10th highest HIV prevalence in the World. Recent estimates are lower, 11.5 per cent [40], but still very high by international standards. In the southern Gaza province, where data for this study were collected, HIV prevalence in 2009 was estimated at 25 per cent [40]. The Republic of South Africa has long served as the pivot of the labor migration system in the southern African region drawing migrants from neighboring countries to its mining sector [41,42]. Labor migration from rural areas of southern Mozambique to the mines and other destinations in South Africa has been an important feature of the area’s economy since the colonial era [43,44]. Mozambique has also witnessed a steady increase in internal migration to its urban centers especially during the period of the civil war (the end of 1970s–1992) when rural residents sought safety in cities. More recently, socioeconomic imbalances amplified by structural adjustment policies, strains on the economy from environmental shocks (such as floods and droughts), erratic and low agricultural yields, scarce non-agricultural jobs and rising cost of living have all contributed to an increase in both internal and, especially, international migration [45].

Reflecting the described labor migration regime and high HIV prevalence has been the heightened notion in southern Mozambique that HIV/AIDS is a disease brought from South Africa by labor migrants [32]. Recent research provides support for this notion as migrant miners were found to have reported risky behavior such as having multiple sexual partners and low condom use in South Africa and at home with their wives [42]. Migrants’ wives risk of infection is further complicated by their inability to insist on condom use as this could be interpreted as questioning their husbands’ fidelity. However, we must be quick to note that these perceptions could be controversial as migrants may contract HIV in their home villages prior to migrating, along the way to and from urban areas in their own country or in their destination in South Africa. Similarly, migrants’ wives have been found to be more likely to engage in extramarital sex than wives of non-migrants [46] and research has also shown that the direction of the spread of AIDS is not only from returning migrant men to their rural partners, but from women to their migrant partners [27].

## 5. Methods

### 5.1. Data

The data used in this study were collected in 2006 as part of a collaborative project by research teams from Arizona State University and Eduardo Mondlane University in Mozambique. A probability sample was drawn among women aged 18–40 residing in 56 villages of four districts of Gaza province in southern Mozambique (with approximately a population of 625,000). In each district, 14 villages were selected with probability proportional to size. In each selected village, all households with at least one married woman were canvassed and recorded into two lists: those with at least one woman married to a migrant (a migrant was defined based on husbands who spent all nights outside the community in the last month for the purpose of supporting their family and thus includes internal and external migrants) and those without such women. These two lists were used as sampling frames; from each list, 15 households were randomly selected. In each selected household a woman was interviewed (in households classified as migrant, a woman married to a migrant was interviewed). This procedure yielded a total sample of 1,680 women (420 per district, 30 per village) of these women 41% were wives of migrants and 59% were wives of non-migrants. 

The survey instrument included questions on a variety of respondents’ sociodemographic characteristics as well as on HIV/AIDS awareness and prevention. A separate module of the survey questionnaire was devoted to respondent’s’ relationship and interaction with personal network members. Due to concerns of how long interviews could last without undue fatigue, each respondent was asked to name at most three people with whom she had most interaction and greatest trust (apart from her spouse and children). Detailed socioeconomic characteristics were then gathered on each personal network member named. In addition to socio-economic data, respondents were asked about HIV/AIDS-related and other health conversations they might have had with their network members and the network members’ HIV prevention and testing behavior in the recent past. Due to the sensitive nature of the data, the survey questionnaire was conducted by an interviewer of corresponding gender in Portuguese, the official language of Mozambique. In a few instances when respondents had limited Portuguese proficiency, interviews were conducted by interviewers with relevant local language skills. Ethical approval for the survey was obtained from Arizona State University Institutional Review Board (IRB) and the collaborating institution obtained the necessary ethical clearances in Mozambique. Fifty-two sampled respondents were not available to interview and only three refused (or were prohibited by their husbands to be interviewed).

The analysis in this study is limited to those with at least one personal network member (less than 1% of the total sample was excluded because respondents did not name any network members). Nearly all network members (98%) were women and on average 2.2 (s.d. 0.80) network members were reported. A bivariate analysis of sociodemographic differences between women who reported 1, 2, or 3 network members did not reveal any biases. However, limiting to three the number of alters on whom detailed information was gathered and obtaining that information from reports by ego limited our analysis and influenced the constructions of our measures in a few ways. First, no meaningful measures of network locations (such as density, size *etc.*) could be constructed to test how such locations facilitate access to network resources and how that impacts on health outcomes and behaviors and second, information on alters’ HIV prevention and testing behavior may be the perception of ego and not the perception or experiences of alters. Lastly, to more effectively use data we utilized the ego-network member dyad as the unit of analysis. For example, if one network member is reported, only one observation is contributed to the analysis, whereas a case in which three members are named contributes three observations. While this approach allows us to use data more effectively, it also creates a problem of within-respondent clustering of observations, as personal network members of the same ego may share some unobserved characteristics. Thus we employ a random intercept model that allows the intercept to vary randomly by respondent to account for the possible correlation between the set of network members of the same respondent. 

### 5.2. Measures

We constructed the dependent variable for the test of our first hypothesis as whether or not a (any) network member is a spouse of a current labor migrant. This variable was derived from responses to the question asking ego if the spouse or partner of their personal network members worked in the community, outside the community or did not work at all. This variable is coded as a dichotomous indicator of whether or not network member’s spouse worked outside the community (*i.e.*, was a labor migrant) *vs.* otherwise.

The dependent variable for the test of our second hypothesis was constructed from responses to the question “Was AIDS ever mentioned in your conversations with ‘network member’, even if briefly?” Even though the question did not specify a time period for conversations, we assume that such conversations, if reported, occurred in the recent past. This outcome is also operationalized as a dichotomy. The test of the third hypothesis also uses this outcome. 

The dependent outcome for the test of our fourth hypothesis is limited to women who mentioned AIDS in their conversations with their network members. These women were asked to describe the content of their most recent conversation about AIDS. Responses to this question included: known AIDS cases, prevention of HIV, testing and treatment of HIV/AIDS, and other themes. Each response category was coded dichotomously and tested separately.

The outcome for the test of the fifth hypothesis was constructed by asking respondents what they were doing in order to protect themselves from contracting HIV and the number of times they had been tested for the disease. Reponses included: doing nothing, using condoms, fidelity to husband, abstinence from sex, avoiding contact with blood or injections and practicing some other forms of prevention. Due to fewer respondents in some categories (e.g., using condoms) we coded this outcome into a dichotomous measure where 1 represented any form or combination of forms of HIV prevention and 0 if otherwise. Similarly, testing for HIV was dichotomously coded with 1 representing respondents who have tested for HIV at least once and 0 if otherwise.

The main independent variable is husband’s labor migration status. This was a dichotomous indicator and was coded 1 if the respondent’s spouse was a labor migrant at the time of the survey and 0 if otherwise. Given that our conceptual framework highlights the effects of heightened fears of HIV infection of migrants’ wives, a second dichotomous independent variable was included to measure whether or not the respondent was very worried or a little worried about the possibility of contracting the AIDS virus from her spouse. The third independent variable of interest was also a dichotomous variable of whether a (any) personal network member was married to a migrant or not (also used as a dependent variable to test hypothesis one as described above).

Two sets of other predictors for the association between network members’ HIV prevention and testing behavior and that of egos were constructed. Egos were asked whether they knew what methods of prevention their network members used to protect themselves from HIV. The response options for this question were identical to those for the question asked of egos themselves (uses condoms, faithful to husband, abstain from sex, other and does nothing) and the variable was coded dichotomously—1 if network member used a (any) form or combination of forms of prevention and 0 if otherwise. Lastly, egos were asked if they knew their network members had done an HIV test. This was also coded dichotomously with 1 indicating that the network member had done a test at least once and 0 if otherwise.

To measure network resources within personal networks that can be accessed and mobilized for health promotion and maintenance, we used information gathered from ego on network members to construct five dichotomous measures of network characteristics and resources: (i) whether or not a (any) network member is kin or non-kin relation of ego; (ii) age of network member defined relative to ego (older than ego, younger than ego or about the same age as ego); (iii) religion of network member also defined relative to ego (same religion as ego or of a different religion); (iv) whether or not network member works outside the household and (v) whether or not network member is willing to loan money to ego in case of urgent need. We argue that these network characteristics can serve as proxies for vital resources available within a personal network and create trust within the network. For example, being of a similar age as one’s network members may enable the sharing of similar interests and resources. Being of the same religion may facilitate communication relevant to health outcomes. 

Lastly, we include as statistical controls standard socioeconomic characteristics of ego that may influence the relationship between migration and HIV/AIDS outcomes. These variables include: ego’s age and number of living children (both defined continuously); education (coded in three categories; 0–4 years of school, 5–7 years of school and 8 or more years of school); employment (works for income or not); type of marriage (monogamous or polygynous union); religious affiliation (coded in three groups, reflecting the religious composition of the predominantly Christian study area: mainline churches, Evangelical and Pentecostal- churches, and none); household material possessions (defined on a 4-level scale: 1. no radio, bicycle, motorcycle, or car; 2. radio, but no bicycle, motorcycle, or car; 3. bicycle but no motorcycle or car; 4. motorcycle or car); type of roof of respondent’s primary dwelling place (thatched *vs.* zinc, polyurethane or block roof) and whether the respondent’s household owns cattle. Also, we controlled for whether migrants’ wives resided in a household with parents-in-law as this may influence the autonomy of wives and consequently AIDS-related networks and behavior. Lastly, a dichotomous indicator of whether or not HIV/AIDS was mentioned in recent conversations with husband was included as it may possibly be associated with HIV/AIDS communication and behavior within personal networks of wives.

### 5.3. Statistical Model

As indicated earlier, the dyad ego-network member was chosen as our unit of analysis. For all statistical text, we employed a random intercept logistic model that allows the intercept to vary randomly by respondent to account for the possible correlation within the set of network members of the same respondent. 

Similarly, the sampling is clustered by village which may result in biased estimates due to the non-independence of women in the same village. To tackle this other source of potential bias, we introduce a second random intercept to account for clustering of respondents within villages. We fit the resulting multi-level random intercept models using the Glimmix procedure in SAS 9.2. 

Although the use of pseudo-likelihood estimation under Glimmix (rather than maximum likelihood estimation) may present additional bias and prevent the use of accurate standard goodness-of-fit measures, it is generally considered a good alternative to, for example, the MIXED procedure in SAS as it allows for several random effects with categorical outcomes [47] and the generalized chi-square and generalized chi-square divided by the degrees of freedom are useful measures in assessing goodness of fit of models [48]. Lastly, having undertaken these techniques to minimize bias in the estimates, we must note that the cross-sectional nature of our data does not allow us to ascertain causality.

## 6. Results

### 6.1. Descriptive Analysis

We begin with the presentation of network characteristics and resources and AIDS- related outcomes by migration status. Table 1 indicates that 42 per cent of the network members were migrants’ wives, compared to 55 per cent who were married to non-migrants’ wives (the remaining 3% were not married). Personal network members were mainly made up of non-kin ties (neighbors, co-workers and friends) as opposed to relatives. This did not differ by migration status. Over 50 per cent of network members were older than the ego, and half were of the same religion. Similarly, a high proportion of network members (86%) were reported to be likely to loan ego money if necessary. On AIDS-related outcomes, migrants’ wives were more likely to have conversed about HIV/AIDS with their personal network members (69%) than non-migrants’ wives (69% *vs.* 62%). There were only slight and not statistically significant differences in network members’ and ego’s use of forms of HIV prevention and testing by spouse’s migration status. None of the variables representing network resources and AIDS-related outcomes, apart from having network members who were married to migrants and having AIDS-related communication, showed statistically significant variation by spouse’s labor migration status.

Table 2 displays the distribution of specific themes of HIV/AIDS-related communication among women who had conversed about HIV/AIDS by husband’s migration status. Following the options in the questionnaire, four main themes of conversations emerged: (1) *AIDS cases (both known and suspected)*; (2) *Need for prevention of AIDS* (3) *Testing for HIV and treatment of AIDS and* (4) *Other themes*. As seen from the table, conversations about prevention dominated AIDS-related communication, followed by discussions of suspected or known cases of HIV/AIDS. Migrants’ wives had higher proportions of those reporting any of the three specific AIDS-related themes. 

**Table 1:** Network members and ego’s characteristics by husband’s labor migration status.

		Husband’s Labor Migration Status		
Characteristic		Migrant	Not a migrant	All
Network member is married to migrant **		42.42	55.3	47.98
Network member is Kin or in-law		37.99	36.31	37.12
Network member’s age relative to ego				
	Older than ego	52.45	50.41	51.37
	Same as ego	19.16	18.86	18.95
	Younger than ego	28.39	30.73	29.68
Religion				
	Same as ego’s	50.5	47.84	48.95
	Other/No religion or don’t know	49.5	52.16	51.05
Network member will loan ego money if in need		86.91	85.18	85.96
Network member works outside the household		12.95	12.16	12.47
Ever talked about AIDS with network member **		69.35	62.07	65.13
Network member uses at least one method of HIV prevention		34.17	32.2	33.06
Network member had an AIDS test		6.76	5.47	6.01
Ego’s uses at least one method of HIV prevention		81.58	79.38	80.1
Ego had AIDS test		18.2	16.77	17.34
Total		42.93	57.07	100
N		1,390	1,848	3,238

** *p* < 0.01. Notes: Number of observations for ego—1,678; number of network dyads—3,238.

**Table 2:** Themes of HIV/AIDS related conversations in social networks.

Husband’s Migration Status			
Themes	Migrant	Not a migrant	All
Need for Prevention	92.22 *	88.23 *	90.07
Known or Suspected AIDS Cases	64.21 *	59.55 *	61.59
Testing and Treatment of AIDS	23.65 *	19.97 *	21.62
Other themes	4.99	4.81	4.89

Notes: More than one theme per partner is possible, percentages do not add up to 100. * *p* < 0.05.

### 6.2. Multivariate Analysis

Odds ratios from multilevel random effect models are presented on Table 3. Each of the three models of the table corresponds with one or two of our hypotheses; for the first model only a main-effect model (including ego’s characteristics as statistical controls) is presented and for the second and third models interaction terms are included. We start by testing the first hypothesis—whether migrants’ wives are more likely to have migrants’ wives as their personal network members, net of other factors. Results of Model 1 indicate that indeed migrants’ wives are significantly more likely to have network members who are also married to migrants, net of socio-economic characteristics of ego. The odds among migrants’ wives of reporting network members who were migrants’ wives were 1.5 times those among non-migrants’ wives. This result provides support for the first hypothesis. In the same model, respondents who express worry about the possibility of contracting the AIDS virus from their spouses was not significantly associated with having network members who are married to migrants.

**Table 3:** Women’s personal network composition and content of communication about HIV/AIDS, odds ratios, multilevel random effect models.

		1. Network member is married to migrant	2. Talked about AIDS with network members		3. Talked about HIV prevention in conversation on AIDS	
			2A	2B	3A	3B
Labor migration						
	Migrant’s wife	1.50 **	1.84 **	1.28	2.17 **	1.52
Worried of AIDS infection from spouse		1.08	3.31 **	2.98 **	2.29 **	2.11 *
Network member is married to migrant			1.44 **	1.33 †	1.19	1.06
Network Resources						
	Kin		0.90	0.90	0.94	0.94
	Older than ego		1.05	1.05	1.01	1.01
	Younger than ego		1.11	1.11	0.98	0.98
	Same religion as ego’s		1.04	1.04	1.10	1.10
	Network member would loan money		1.19	1.17	1.32	1.30
	Network member works		1.23	1.24	1.16	1.18
Ego’s characteristics						
	Age (in years)	0.98 *	1.04 *	1.04 *	1.03	1.02
	Number of living children	0.98	1.00	1.00	1.060 *	1.06
	1–4 years of school	1.08	1.36	1.37	1.19	1.19
	5 or more years of school	1.26 †	2.65 **	2.71 **	1.74 *	1.91 *
	Currently working	0.83 †	2.00 **	2.01 **	1.72 *	1.73 *
	In polygynous union	0.86	1.23	1.23	1.5 *	1.50 *
	Resides with parents in-law	1.03	1.06	1.06	1.04	1.04
	Household material possession index	1.09 †	1.01	1.00	1.02	1.02
	Thatched roof	0.86 †	1.00	1.00	0.94	0.94
	Household own cattle	1.04	1.12	1.13	0.99	0.99
	Mainline church	1.09	1.30	1.30	1.39	1.39
	Zoinist/Pentecostal	0.95	1.69 *	1.69 *	1.64 *	1.64
	Had talked to husband about AIDS	1.15	9.77 **	9.82 **	8.785 **	8.81 **
Migrant’s wife*worried of AIDS infection from spouse				1.37		1.29
Migrant’s wife*network member is married to migrant				1.21		1.32
Generalized Chi-square		2,922.0	1,263.7	1,261.00	1,390.0	1,387.33
Generalized Chi-square/DF		0.91	0.40	0.40	0.44	0.44
N		3,227	3,210	3,210	3,210	3,210

Reference categories: Non-migrant’s wife; Does not worry of AIDS infection from husband; Network member is not married to migrant; Non-kin; Same age as ego; Different religion from ego’s; Network member would not loan money; Network member does not work; No education; Not working; In monogamous union; Does not reside with parents in-law; Zinc, polyurethane or block roof; Does not own cattle; No religion; Has not talked to husband about AIDS; Significance level: ** *p* < 0.01; * *p* < 0.05; † *p* < 0.10.

The odds ratios from the model testing the second hypothesis are presented in Model 2a. In this model, we compare the likelihood of HIV/AIDS communication in personal networks of wives of migrants and non-migrants given network characteristics and resources. We observe that migrants’ wives were significantly more likely to have talked about HIV/AIDS with their network members than did non-migrants’ wives, net of network resources and the socio-economic characteristics of ego. The odds of HIV/AIDS-related conversations in personal networks of migrants’ wives are 1.8 times those in personal networks of non-migrants’ wives. In that model, respondents who express worry about the possibility of contracting the AIDS virus from their spouses were significantly more likely to communicate about HIV/AIDS with their network members. The odds of HIV/AIDS communication for women who express worry are 3.3 times that of those who did not. Our third key predictor, whether network members were married to labor migrants, was also significantly associated with HIV/AIDS conversations (OR = 1.41). This effect reinforces not only our first hypotheses but offers partial support for our second hypothesis that migrants’ wives are more likely to converse about HIV/AIDS than non-migrants’ wives. However, although pointing in the expected direction, none of the variables representing network resources was significantly associated with HIV/AIDS communication. 

For the test of the third hypothesis (Model 2B), we include an interaction term to ascertain whether migrants’ wives are more likely than non-migrants’ wives to converse about HIV/AIDS with their fellow migrants’ wives. In that model, we also include an interaction term between spouse migration status and women’s expression of worry about contracting the AIDS virus from their spouses. Results from Model 2B show that the variable for spouse labor migration status which was significantly associated with HIV/AIDS communication in Model 2A is no longer statistically significant, while that of women who express worry about infection from husbands remains highly significant net of network resources and other characteristics (OR = 2.98). Similarly, both interaction terms were found not statistically significant. First, this implies that even though migrants’ wives and their personal network members who were also married to migrants are more likely to converse about HIV/AIDS, they are no more likely to seek out other migrants’ wives for such conversations than non-migrants’ wives. Hypothesis three is therefore not supported. Second, migrants’ wives who express worry about getting AIDS from their husbands are no more likely to talk about AIDS than non-migrants’ wives who express the same worry. Thus AIDS communication seems to be predicated on worry of getting infected by spouse irrespective of whether that spouse is a migrant or a non-migrant. 

We then consider themes of AIDS-related conversations. Although at the bivariate level we saw a statistically significant difference by husband’s migration status in reporting conversations about known AIDS cases, this difference became non-significant in the multivariate test (not shown). Similarly, the multivariate tests did not detect any significant differences in discussions on HIV testing and treatment of HIV/AIDS (not shown). The only statistically significant variations by migration status were in conversations that revolved around HIV prevention. Thus in Model 3A, we observe that migrants’ wives were significantly more likely to have talked about HIV prevention in their AIDS-related communication than were non-migrants’ wives: the odds of migrants’ wives mentioning prevention in AIDS conversations were more than twice those of non-migrants’ wives net of network resources and other characteristics. These results lend partial support to our fourth hypothesis for although migrants’ wives were more likely to discuss prevention of AIDS than non-migrants’ wives, none of the network characteristics and resources were significantly associated with such discussions. Similarly, respondents who expressed worry of contracting HIV infection from husbands were significantly more likely to discuss HIV prevention in their AIDS conversations than those who did not (OR = 2.3). Notably, the effect of network member being married to a migrant was not significant.

In Model 3B we include the two interaction terms included in Model 2B. Both interaction terms were not statistically significant. Migrants’ wives were no more likely than non-migrants’ wives to discuss HIV prevention with network members who were also married to migrants nor were migrants’ wives who express worry of infection more likely to discuss HIV prevention in their conversations than non-migrants’ wives who express the same worry. However, consistent with Model 2B, the variable for spouse labor migration status which was significantly associated with discussion of AIDS prevention in Model 3A is no longer statistically significant, while that of women who express worry of AIDS infection from husbands remains statistically significant, net of network resources and ego’s characteristics. 

**Figure 1:**
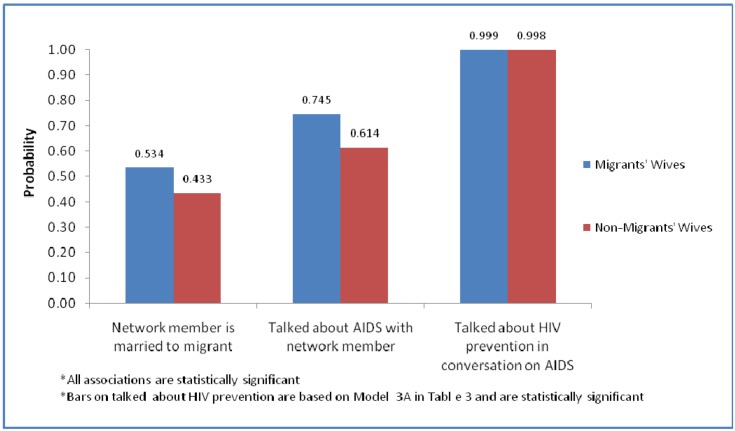
Predicted probabilities of having network members who are married to migrants, AIDS conversations and conversation about HIV prevention.

To more easily grasp our main findings as presented on Table 3, Figure 1 plots the predicted probabilities of our key outcomes (having network members who are married to migrants, having AIDS conversations with network members and talking about prevention in AIDS conversations) by labor migration status. Although Figure 1 is meant to provide visualization of our main findings it should be interpreted in conjunction with the statistical estimates presented in Table 3. Overall, Figure 1 shows that personal network composition and content of conversation of on AIDS seems to be organized around labor migration. Migrants’ wives as echoed by Table 3 were significantly more likely to have migrants’ wives as network members and to talk about AIDS and HIV prevention in those conversations. However, as seen on Table 3, these differences disappear when an interaction between migration status and general worry about AIDS infection from spouse is accounted for. Thus migrants’ wives who express worry of infection from spouse are not significantly different in AIDS outcomes from non-migrants’ wives who express the same worry. 

Lastly, Table 4 presents results of the test of our last hypothesis that migrants’ wives will be more likely to use prevention and be tested for HIV if their network members are also migrants’ wives and have used prevention and tested for HIV. This hypothesis was tested by including interaction terms to ascertain if there is a significant association between the migration status of network members and their use of prevention and testing for HIV and ego’s use of prevention and testing. However, as indicated in Table 4 the test did not detect any statistically significant associations between network member’s migration status and use of HIV prevention and testing and ego’s prevention and testing. Nor did we find any significant association between ego’s spouse migration status and her use of prevention and testing as indicated on Table 4. Thus hypothesis five was not supported.

**Table 4:** Ego’s use of HIV prevention and testing. odds ratios, multilevel random effects models.

		Ego’s Uses HIV Prevention	Ego has Tested for HIV
Labor migration status			
	Migrant’s wife	1.09	1.03
Worried of AIDS infection from spouse		1.34	1.47
Network member is married to migrant		0.76 †	0.93
Network member uses HIV prevention		4.92 **	
Network member has tested for HIV			8.64 **
Network Resources			
	Kin	0.77	1.17
	Older than ego	0.93	0.89
	Younger than ego	1.01	0.99
	Same religion as ego’s	1.27	0.92
	Network member would loan money	1.35	0.96
	Network member works	1.13	1.11
Ego’s characteristics			
	Age (in years)	1.01	0.96 *
	Number of living children	0.96	1.17 *
	1–4 years of school	1.15	0.82
	5 or more years of school	1.33	1.43
	Currently working	1.64 *	0.72
	In polygynous union	0.75	1.11
	Resides with parents in-law	0.91	1.01
	Household’s material possession index	1.23 *	1.18
	Thatched roof	1.30	1.07
	Household owns cattle	0.68 †	1.04
	Mainline church	1.01	1.73
	Zionist/Pentecostal	1.09 *	1.97 *
	[No religion]	2.09	1.34
	Had talked to husband about AIDS	2.09 **	1.34
Migrant’s wife*network member is married to migrant*network member prevent HIV		2.10	
Migrant’s wife*network member is married to migrant*network member Tested for HIV			0.60
Generalized Chi-square		903.78	908.50
Generalized Chi-square/DF		0.28	0.29
N		3,210	3,210

Reference categories: Non-migrant’s wife; Does not worry of AIDS infection from husband; Network member is not married to migrant; Network member does not use prevention; Network member has not tested for HIV; Non-kin; Same age as ego; Different religion from ego’s; Network member would not loan money; Network member does not work; No education; Not working; In monogamous union; Does not reside with parents in-law; Zinc, polyurethane or block roof; Does not own cattle; No religion; Has not talked to husband about AIDS; Significance level: ** *p* < 0.01; * *p* <0.05; † p < 0.10.

However, we observe in Table 4 that accounting for individual and network characteristics, egos whose network members report any form of HIV prevention were more likely to use at least one form of prevention themselves. The odds of ego using any form of prevention if her network members also uses were nearly five times compared to those whose network members did not use prevention. Similarly, egos who reported that their personal network members had already tested for HIV were themselves much likely to have been tested for HIV than those who did not (OR = 8.64). However, these results must be interpreted with caution as our data preclude testing for the direction of this association as it is also possible that egos may influence their social network members’ prevention testing behavior or both processes may occur simultaneously. Moreover because our data are based on ego’s perception of alters’ prevention behavior and not the perception or experiences of alters themselves, firm conclusions may not be reached on social influence. 

## 7. Discussion and Conclusions

Incorporating network approaches in research on social capital and health is essential in understanding the pathways through which social capital impact health outcomes. However, the literature on social capital and health has been predominantly conceptualized in the context of community resources such as social cohesion, trust and exercise of sanctions *etc.* rather than in the context of actual and potential resources embedded within personal networks. Similarly, the literatures on social capital and health and migration and HIV/AIDS have remained largely separate. This study was designed to help fill these gaps by developing and testing specific hypotheses on how left-behind women in rural Mozambique use resources within their personal networks to cope with the risk of HIV infection and the role of homophily, selection, and social influence in that process. Although due to the cross-sectional nature of the data, the detected associations cannot be interpreted in causal terms, these associations are nonetheless illuminating. 

Our findings should be interpreted in light of the literatures on social capital and health on one hand, and research on personal networks and reproductive and sexual behavior, on the other. First, our finding that migrants’ wives were more likely to have fellow migrants’ wives as personal network members indicates that the choice of network members among stay-behind women in southern Mozambique may be homophily-driven. This aligns with studies on social capital and health outcomes (such as psychological distress, smoking, and alcohol use) that have demonstrated that homophily based on demographic characteristics (such as age, race/ethnicity, sex, and education) and genetic-related traits (such as appearance, intelligence and personality) are crucial in the formation of friendship ties [49], which, in turn, influence health behavior. Similarly, our findings on homophily are consistent with network social capital concepts of closure or density [1,50]. Dense networks have been found to maintain and reproduce group solidarity thereby making it possible to mobilize network resources [1]. However, unlike previous findings, homophilous networks of migrants’ wives in this study did not necessarily generate a better return on HIV/AIDS outcomes as migrants’ wives although talked more about AIDS they did not engage in AIDS discussions with fellow migrants’ wives any more than non-migrants’ wives. Nor did they take steps to prevent or test for HIV any more than non-migrants’ wives. These results indicate that homophily may not be beneficial in AIDS networks and indeed given the advanced stages of the HIV epidemic in sub-Saharan Africa, accessing and extending bridges (as suggested by [51,52] or possessing a more sparse network may be what is required in order to obtain more information and resources necessary to avoid HIV infection. 

Furthermore, our conceptual framework and hypotheses hinged on how variations due to worry about HIV infection surrounding labor migration may affect access to network resources which can be mobilized to maintain physical health. However, our results failed to fully support that conceptualization given that, although pointing in the expected direction, the associations of network characteristics and resources with AIDS outcomes were not significant once migration status and worry about infection from spouse were controlled. Thus, distribution of resources embedded in personal networks did not vary by labor migration and had no significant effect on AIDS outcomes. Rather, AIDS communication seems to be organized mainly around worry of contracting HIV infection from spouses in general and not just limited to migrants. This might further support our suggestion that the generalized nature of the HIV epidemic in sub-Saharan Africa makes the mobilization of such resources not necessarily contingent on labor migration status. Furthermore, although Bourdieu’s conceptualization cautions against exclusionary principles of social capital, such effects were not detected by labor migration status. 

Second, by finding statistically significant associations between labor migration on one hand and worry about getting HIV infection from spouses on the other, and conversations about HIV/AIDS and specifically the discussion of HIV prevention in these conversations, we offer further evidence in support of the growing literature on personal networks and reproductive and sexual behavior that highlights the importance of social ties and interconnectedness in dealing with risk perceptions and worries about contracting HIV/AIDS [53,54]. HIV/AIDS discussions within informal networks are selective on labor migration status of ego’s and network member’s husband and on worry about infection in general. Within these networks, members share information, assess their risk of infection and gain social support which may help them to cope with their worries. 

Third, although not directly related to migration, our finding that network members’ HIV prevention and testing behavior is similar to that of ego’s may provide some further evidence of the role of homophily in health behavior in general. This finding is also well aligned with the literature on social capital and health [11,18] and on personal networks and reproductive and sexual behavior [35,36]. Although it is impossible, given our data, to distinguish if this relationship is due to influence or selection, the fact that both bodies of literature assert the importance of perceived behavior or social support from members of personal networks even if these perceptions are inaccurate provide some utility for our findings. Thus, even if ego’s perception of their network member’s prevention and testing behavior is inaccurate or not based on the perception and actual experiences of their network members, it could still be influential in the actual prevention behavior of egos who are worried about HIV infection.

In closing, our findings have some important policy implications and recommendations. First, considering evidence from previous research demonstrating that interpersonal health communication is predictive of preventive behavior such as condom use [55,56], our findings are particularly valuable to programs and policies geared towards combating the HIV/AIDS epidemic. The worry of being infected by migrant spouses (or as suggested by previous research-the risks of returning migrants being infected by their stay-behind wives) offers outreach avenues to target not only migrants’ wives in places of migration origin but also women in general with AIDS communication and intervention programs about prevention. These could be more effectively channeled through personal networks not only as a cost effective means of generating and disseminating accurate information on AIDS but because social capital generated through social networks have been shown to have several powerful effects on health outcomes. Consequently, the international public health community could encourage migrants’ wives (and non-migrants wives) who remain at home to form informal groups for the purpose of disseminating information on prevention of AIDS. 

Second as local and international agencies and governments devote resources to improving testing and treatment facilities and to producing and disseminating anti-retroviral drugs in rural communities, stigma and misunderstanding that still surround the disease must be addressed in order to more effectively respond to the African AIDS crisis. Indeed stigma is often cited as a formidable barrier to accessing prevention, care and treatment services. Yet efforts to combat stigma have been relegated to the bottom of AIDS program priorities [57]. Paying heed to personal networks of migrants’ wives and deliberately training peer educators and front-line health workers to build and foster healthy social relationships within these networks in local communities may help reduce general stigma surrounding the disease and labor migration.

Lastly, addressing deeply ingrained gender norms about sexual behavior and attitudes in migrant communities will contribute to a compressive strategy to combat AIDS. Sexual attitudes and behavior associated with male condom use both within and outside of marriage should not be limited to places of migration destination but should extend to places of migration origin. Married men and women should be targets of such efforts. Using personal networks as avenues for communicating, deliberating and understanding gender norms that put people at risks of HIV may facilitate the long process of changing HIV risks behavior and help meet public health’s most pressing challenges in rural Mozambique and similar sub-Saharan settings. 
